# Research on strength prediction model of manufactured sand‌ concrete during steam curing stage based on equivalent age

**DOI:** 10.1038/s41598-026-48161-7

**Published:** 2026-04-11

**Authors:** Fan Li, Xiang Gao, Dongcun Huo, Lili Han, Kangjia Fan, Yun Duan

**Affiliations:** 1 School of Highway Engineering, Shaanxi College of Communications Technology, Xi’an, Shaanxi China; 2 High-speed Rail Engineering College, Shaanxi Railway Institute, Weinan, Shaanxi China; 3 National Local Joint Engineering Laboratory for Disaster Prevention and Control Technology in Road and Bridge Engineering, Lanzhou Jiaotong University, Lanzhou, China

**Keywords:** Prefabricated components, Manufactured sand concrete, Steam curing, Compressive strength, Prediction model, Engineering, Materials science, Mathematics and computing

## Abstract

To determine the optimal steam curing duration for precast manufactured sand‌ concrete components and the corresponding strength prediction model at this stage, this paper investigates the strength development law of manufactured sand‌ concrete with age under steam curing temperatures of 40 ℃, 50 ℃ and 60 ℃, and discusses the compressive strength prediction model of manufactured sand concrete at the steam curing stage based on the theory of maturity at the equivalent age of Arrhenius. The results show that steam curing can significantly shorten the time required for manufactured sand‌ concrete to reach the design strength, but the temperature effect of steam curing is limited and the curing duration should not be excessively long. Specifically, the steam curing duration at 60 ℃ should not exceed 12 h, at 50 ℃ should not exceed 24 h, and at 40 ℃ should not exceed 48 h. Among the prediction models based on equivalent age, the hyperbolic function prediction model has the highest accuracy, while the accuracy of the exponential function prediction model is the lowest. For the strength prediction of manufactured sand‌ concrete at steam curing temperatures of 50 ℃ or below, it is suitable to choose the hyperbolic function calculation model.

## Introduction

Natural river sand resources are becoming increasingly scarce, and manufactured sand, as a substitute for river sand, is gradually replacing river sand in engineering, becoming the main source of material for construction sand. Manufactured‌ sand is produced by mechanical crushing, and fine particles with particle size of less than 75 μm, known as stone powder. Li et al.^1^ showed that the appropriate amount of stone powder in manufactured sand is conducive to improving the packing density of manufactured sand and concrete workability; on the contrary, an excessively low stone powder is likely to cause concrete segregation and bleeding. The hydrophilicity of stone powder from different lithologies varies, and its effect on the workability of the mixture differs with increasing content, which leads to the difference in the optimal content of stone powder for manufactured sand concrete^2–5^. Stone powder also serves to densify the interfacial transition zone and improve the pore structure of concrete, leading to a certain degree of pore refinement and thus enhancing the mechanical properties of manufactured sand concrete. Li et al.^6^, Fan et al.^7^, Zhao et al.^8^, Sun et al.^9^, and Anitha et al^10^. investigated the effects of varying stone powder contents on the mechanical properties of concrete with different strength grades. They revealed the influence laws of stone powder content on concrete strength and durability, and proposed the optimal stone powder content for concrete respectively. Li et al.^11^, Li et al.^12^, Zhang et al.^13^ and other scholars found that an appropriate amount of stone powder can densify the internal structure of manufactured sand concrete and enhance its impermeability, and it also exerts a certain effect on the freeze-thaw resistance of concrete.

With the rapid development of highway and railway construction, precast components are in increasing demand in the industry owing to their excellent quality control, Therefore, producing low-cost, high-performance components within a short time has become the focus and challenge in engineering. Steam curing enables concrete to achieve the design strength in a short time, significantly improves the utilization efficiency of precast component production sites and formwork, and has become the most widely used curing method for precast components. Kim et al.^14,15^ found that high-temperature steam curing could effectively enhance the early-age compressive strength and splitting tensile strength of concrete. Zhang et al.^16^ revealed a positive correlation between steam curing temperature and the development of early-age concrete strength. It is suggested that the steam curing temperature should be controlled within the range of 65 °C to 85 °C for higher early-age strength, and should not exceed 90 °C; otherwise, it would lead to a reduction in the long-term strength of concrete^17^. Zhang et al.^18^ tested the concrete strength data under different curing regimes, analyzed the effects of pre-curing time, heating rate and cooling rate on the demoulding compressive strength and 28-day compressive strength of concrete, and established prediction models for the demoulding compressive strength and 28-day compressive strength of concrete. He et al.^19^ predicted the hydration exothermic curves of steam-cured concrete based on the equivalent age theory. The results indicated that a good linear correlation existed between the cement hydration heat release and the compressive strength of steam-cured concrete, and the compressive strength of steam-cured concrete at the end of the isothermal stage could reach 60% or more of its 28-day compressive strength. Chen et al.^20^ investigated the effects of curing temperatures (20 ℃/40 ℃/60 ℃/80 ℃), steam curing durations (6 h/9 h/12 h) and mineral admixtures (slag powder and fly ash) on the demoulding strength and strength development of recycled aggregate concrete (RAC). Based on the Arrhenius equation, the hydration reaction rate was characterized by the development of compressive strength, the apparent activation energy associated with steam curing duration was calculated, and an energy-based compressive strength model was established. Gu et al.^21^ adopted four machine learning models—SVM, RF, XGBoost and CNN—to predict the strength of steam-cured concrete. The results demonstrated that the XGBoost model yielded the best strength prediction performance, with the R^2^ and MSE values reaching 0.954 and 18.03, respectively. While steam curing can significantly enhance the early mechanical strength of concrete, adverse effects such as thermal damage, shrinkage cracking, and delayed ettringite formation (DEF) may lead to a slower development of long-term strength or even induce performance degradation^22–26^. Moreover, a higher curing temperature increases the apparent density of C-S-H gel in concrete, which impairs the spatial filling of the cement matrix, leading to increased capillary porosity and a reduction in the long-term compressive strength^27,28^. Shang et al.^29^ studied the effect of stone powder content on steam-cured concrete and found that the strength of manufactured sand concrete with a high stone powder content develops faster than that with a low content. Liu et al.^30^ found that replacing river sand with manufactured sand can effectively alleviate the thermal damage caused by the steam curing. Compared with steam-cured river sand concrete, steam-cured manufactured sand concrete exhibits superior mechanical properties and better microstructure. Liu et al.^31^ investigated the time-dependent mechanical properties of fly ash-slag blended manufactured sand concrete under steam curing at a constant temperature of 45 ℃. They found that under the steam plus standard curing regime, the time-varying model for the compressive strength of concrete can be expressed as a logarithmic function or a composite function of logarithm and power function, while those for the splitting tensile strength and elastic modulus can be represented by a power function.

At present, most studies on the strength of steam-cured concrete have focused on natural sand concrete, with only a small number of investigations targeting manufactured sand concrete. Existing strength prediction models based on the theories of maturity, equivalent age and activation energy mainly focus on describing the time-dependent strength development of concrete under steam curing conditions, and have achieved satisfactory performance in ordinary concrete or recycled aggregate concrete. However, for manufactured sand concrete with high stone powder content, more complex aggregate surface characteristics and interfacial interactions, the applicability of parameters in the above models and their ability to characterize the coupling effect of steam curing temperature and duration have not yet been systematically verified. In addition, most existing studies concentrate on the influence of steam curing regime on strength development, while there remains insufficient research on the quantitative determination of the optimal steam curing duration at different steam curing temperatures, as well as its relationship with equivalent age parameters for manufactured sand concrete of the strength grades commonly used in precast components.

Accordingly, this study conducted steam curing tests on C50 manufactured sand concrete, which is widely used in bridge prefabricated components. The dependence between steam curing duration and compressive strength was analyzed, and the optimal curing duration under different steam curing temperatures was determined. Furthermore, the compressive strength of concrete during the steam curing stage was predicted based on the Arrhenius equivalent age theory. On this basis, the applicability of the equivalent age model to steam‑cured C50 manufactured sand concrete was further evaluated, and the influence mechanism of steam curing temperature and duration on early‑age strength development was revealed. The results are expected to provide a reference for determining the reasonable steam curing temperatures and durations for manufactured sand concrete in engineering practice.

## Maturity theory based on equivalent ages

The development of concrete compressive strength is positively correlated with the degree of cement hydration, which depends on the hydration rate and curing duration. The hydration rate is governed by temperature, indicating that concrete strength development is a function of the combined effects of time and temperature^32^.

### Equivalent age based on Arrhenius

The Arrhenius formula describes the relationship between the chemical reaction rate and temperature. Since cement hydration in concrete is also a chemical reaction process, Arrhenius’ law is applicable to describing the cement hydration process, as follows^33^

1 $$K=A\exp \frac{{ - E}}{{RT}}$$

where K is the rate constant of the chemical reaction, A is the Arrhenius constant, E is the activation energy (J/mol), R is the molar gas constant (J/mol-K), and T is the absolute temperature (K).

From Eq. (1), the chemical reaction rate at any two temperatures can be expressed as

2 $$K\left( T \right)=\frac{{{K_1}}}{{{K_2}}}{\mathrm{=}}\exp \frac{E}{R}\left( {\frac{1}{{{T_2}}} - \frac{1}{{{T_1}}}} \right)$$

where K_1_ is the chemical reaction rate constant at temperature T_1_, K_2_ is the chemical reaction rate constant at temperature T_2_, and T_1_ and T_2_ are the absolute temperatures at any two moments (K).

When the chemical reaction rates are identical at T_1_ and T_2_ temperatures, the relationship between the required time is as follows

3 $${K_1} \times {t_1}={K_2} \times {t_2}$$

where t_1_ is the elapsed time at temperature T_1_ (h), t_2_ is the elapsed time at temperature T_2_ (h).

From Eq. (3), when the chemical reaction rate at the actual temperature T_r_ is equal to that at the reference temperature, the corresponding equivalent elapsed time can be expressed as

4 $${t_e}{\mathrm{=}}\int_{0}^{t} {\exp \frac{E}{R}\left( {\frac{1}{{{T_{\mathrm{a}}}}} - \frac{1}{{{T_r}}}} \right)} dt$$

where t_e_ is the equivalent age at the reference temperature T_a_ (h), t is time (h), T_r_ is the actual temperature (K), T_a_ is the reference temperature (K), E is the apparent activation energy of concrete (kJ/mol), and R is the universal gas constant (J/mol·K).

### Concrete strength and age relationship

The relationship between the compressive strength of concrete and its curing age is commonly characterized by exponential (Exp), hyperbolic (Hyp), and logarithmic (Log) functions^34–36^, as expressed below:

5 $$S{\mathrm{=}}{S_u} \cdot \exp {\left[ { - \left( {{\tau \mathord{\left/ {\vphantom {\tau t}} \right. \kern-0pt} t}} \right)} \right]^\alpha }$$

6 $$\frac{S}{{{S_u}}}=\frac{{k \cdot \left( {t - {t_0}} \right)}}{{1+k \cdot \left( {t - {t_0}} \right)}}$$

7 $$S{\mathrm{=}}a+b \cdot \log {}_{{10}}\left( t \right)$$

where S is the compressive strength at age t (MPa), S_u_ is the ultimate compressive strength (MPa), t is the curing age (d), τ is the time constant (d), and α is the shape constant; k is the rate constant (d^− 1^), t_0_ is the time at which strength begins to develop (d), and a and b are the fitting parameters.

## Experimental design

### Raw materials and mix proportion design

P.O 42.5 ordinary Portland cement was used in the test, and its main properties are listed in Table 1. The coarse aggregate was 5–20 mm graded gravel with a crushing value is 5.4%. Fine aggregate was manufactured sand with a fineness modulus of 3.2, stone powder content of 5.6%, methylene blue (MB) value of 0.8, and a loose bulk density of 1533 kg/m^3^.

**Table 1:** Cement properties.

Indicator numerical	Value
Loss on burn (%)	3.31
Fineness (%)	1.40
Specific surface area (m^2^/kg)	351
Alkalinity content (%)	1.04
SO_3_ (%)	2.30
3 d Flexural strength (MPa)	5.03
3 d Compressive strength (MPa)	22.28

Bridge girders in highway and railway construction are generally designed with C50 concrete. Therefore, the concrete mix proportion in this study was designed for C50 grade, as shown in Table 2.

**Table 2:** The mix proportion of concrete.

Water/(kg/m^3^)	Cement /(kg/m^3^)	Coarse aggregate /(kg/m^3^)	Fine aggregate /(kg/m^3^)	Water reducer/%
156	485	1157	680	1.2%

### Specimen preparation and curing system

To reduce the influence of initial temperature on concrete strength development, the concrete batches under each curing regime were mixed in the same test environment, and the variation in ambient temperature before and after the test was controlled within 2 ℃. The compressive strength specimens were 100 mm×100 mm×100 mm cubes, and three specimens were tested for each curing age. PT100 temperature sensors were used to measure the internal temperature of concrete specimens.

The adoption of relatively low steam curing temperatures (45–60 ℃) for a longer duration (24 h) yields better performance in both early and later ages compared with higher temperatures and shorter curing durations^17^. Accordingly, in this study, concrete specimens were prepared under three steam curing regimes at 40 ℃, 50 ℃, and 60 ℃, with standard curing as the control group. Steam curing comprises four stages: pre-curing, heating, isothermal curing, and cooling. The pre-curing duration and heating/cooling rates were identical among all regimes. Pre-curing was conducted for 4 h at 20 ± 2 ℃, and both the heating and cooling rates were 10 ℃/h. Mindess^37^ pointed out that a longer steam curing duration (24 h) results in a higher compressive strength at the early age of 1 day compared with a shorter duration (12 h). However, an extended curing duration exerts an adverse effect on the compressive strength at a later age of 90 days. Therefore, to determine the optimal steam curing regime, this study investigated the compressive strength of manufactured sand concrete at seven constant-temperature curing durations: 3, 6, 12, 24, 48, 72 and 96 h.

### Compressive strength testing program

The compressive strength of manufactured sand concrete was tested in accordance with the Standard for Test Methods of Physical and Mechanical Properties of Concrete. Under standard curing conditions (20 ± 2 °C, RH ≥ 95%), the test ages were set as 3, 7, 14 and 28 days of constant-temperature curing.

## Analysis of test results

### Analysis of concrete compressive strength

**Fig. 1:**
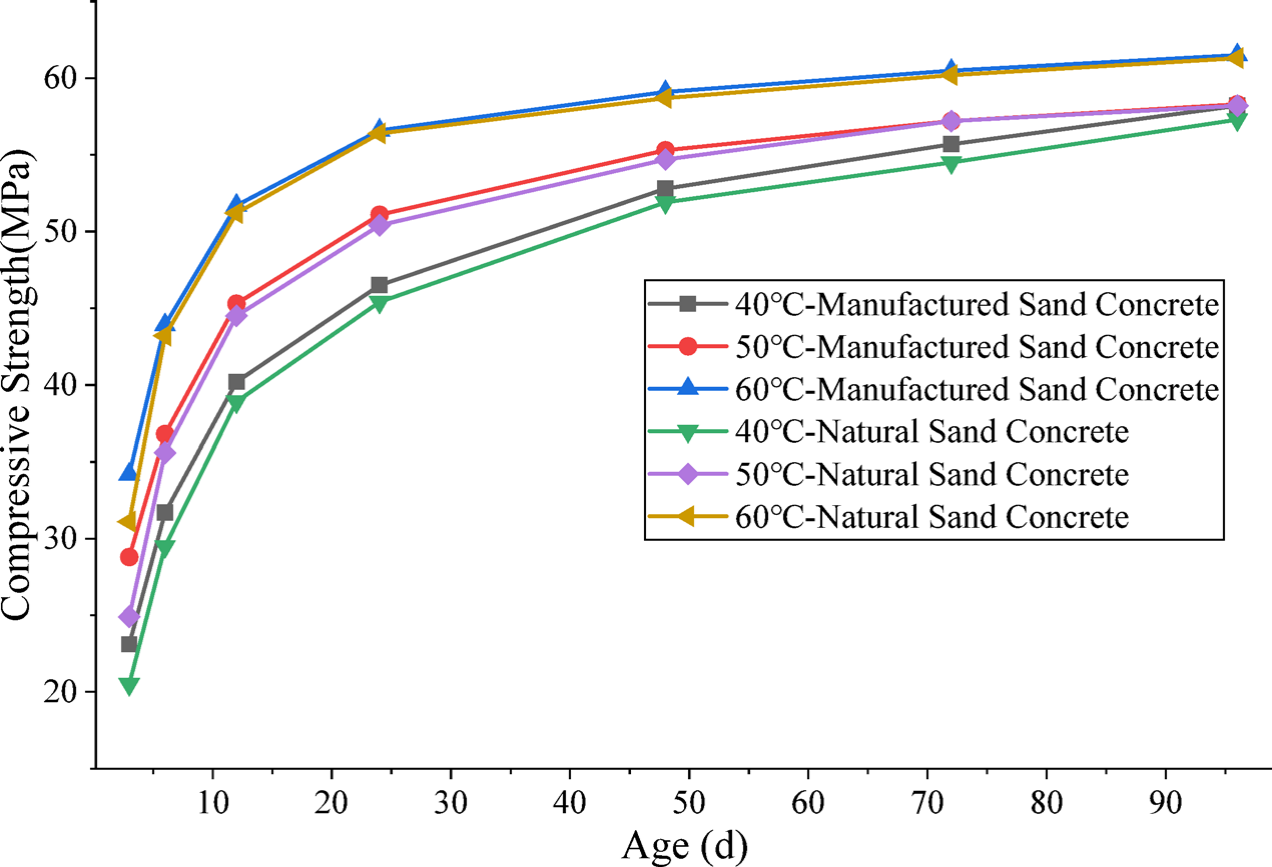
Relationship between concrete strength and age under steam curing.

The compressive strength test results under different curing regimes are presented in Tables 3 and 4; Fig. 1.

**Table 3:** Compressive strength of manufactured sand concrete at different temperatures.

Standard (20℃) conservation		Constant temperature duration/h	40℃ conservation			50℃ conservation			60℃ conservation		
Age/d	Strength/MPa		Age/h	Age/d	Strength/MPa	Age/h	Age/d	Strength/MPa	Age/h	Age/d	Strength/MPa
3	47.5	3	9	0.38	23.1	10	0.42	28.8	11	0.46	34.2
7	53.6	6	12	0.50	31.7	13	0.54	36.8	14	0.58	43.9
14	59.1	12	18	0.75	40.2	19	0.79	45.3	20	0.83	51.7
28	60.9	24	30	1.25	46.5	31	1.29	51.1	32	1.33	56.6
–	–	48	54	2.25	52.8	55	2.29	55.3	55	2.33	59.1
-	–	72	78	3.25	55.7	79	3.29	57.2	80	3.33	60.5
–	–	96	102	4.25	58.2	103	4.29	58.3	104	4.33	61.5

**Table 4:** Compressive strength of natural sand concrete at different temperatures.

Standard (20℃) conservation		Constant temperature duration/h	40℃ conservation			50℃ conservation			60℃ conservation		
Age/d	Strength/MPa		Age/h	Age/d	Strength/MPa	Age/h	Age/d	Strength/MPa	Age/h	Age/d	Strength/MPa
3	46.1	3	9	0.38	20.5	10	0.42	24.9	11	0.46	31.1
7	52.8	6	12	0.5	29.5	13	0.54	35.6	14	0.58	43.2
14	58.5	12	18	0.75	38.9	19	0.79	44.5	20	0.83	51.2
28	60.4	24	30	1.25	45.4	31	1.29	50.4	32	1.33	56.4
–	–	48	54	2.25	51.9	55	2.29	54.7	56	2.33	58.7
–	–	72	78	3.25	54.5	79	3.29	57.2	80	3.33	60.2
–	–	96	102	4.25	57.3	103	4.29	58.2	104	4.33	61.3

Table 4 presents the variation law of the compressive strength of natural sand concrete with the same mix proportion. By comparing Tables 3 and 4, it can be seen that the early strength of steam‑cured manufactured sand concrete is higher than that of natural sand concrete, which is consistent with the findings of existing studies on the mechanical properties of steam-cured manufactured sand concrete^30^. On the one hand, the stone powder in manufactured sand has a certain micro-aggregate filling effect, thereby rendering the internal structure more dense. On the other hand, the stone powder particles in manufactured sand provide additional nucleation sites for hydration products, thus promoting the formation of early hydration products and further enhancing the early compressive strength.

From Table 3, under steam curing at 40 ℃, manufactured sand concrete reaches the design strength after 48 h (2.25 d) at constant temperature. Under steam curing at 50 ℃, it achieves the design strength after 24 h (1.29 d). At 60 ℃, manufactured sand concrete attains its design strength after only 12 h (0.83 d). In contrast, under standard curing at 20 ℃, it takes 7 days to reach the design strength. The results demonstrate that steam curing can greatly shorten the time required for manufactured sand concrete to achieve its design strength.

It can be observed from Fig. 1 that after the manufactured sand concrete reaches its design strength under the three steam curing regimes, the slopes of the strength development curves decreases significantly, indicating that the effect of steam curing temperature on strength development becomes negligible at this stage. In consideration of the design strength requirements of steam-cured concrete as well as the principles of resource and cost conservation, the steam curing duration for manufactured sand concrete should not be excessively long. For C50 concrete, the steam curing duration should not exceed 12 h at 60 °C, 24 h at 50 °C, and 48 h at 40 °C. These durations can serve as a reference for determining the reasonable steam curing time for manufactured sand concrete.

It can also be found from Fig. 1 that within 12 h of curing, the compressive strength of manufactured sand concrete cured at 50 °C is over 10% higher than that of the concrete cured at 40 °C. The strength difference peaks at 3 h (approximately 24.7%) and gradually decreases with increasing curing age thereafter. A similar trend is observed in the compressive strength difference between the 60 °C and the 50 °C steam curing regimes. The reason is that cement hydration is an exothermic chemical reaction, and temperature can accelerate the reaction rate. A higher initial temperature in the constant-temperature stage leads to a faster hydration rate and a more rapid increase in concrete compressive strength^17^. However, the accelerating effect of temperature on cement hydration is not sustainable indefinitely. At 48 h, the difference in compressive strength between these two steam curing temperatures is only 4.7%, indicating that the effect of steam curing temperature is time-dependent.

Further extension of the isothermal curing duration shows that the compressive strength of concrete cured at 60 ℃ is lower than that cured at 50 ℃. This is consistent with the findings of He et al. and Şemsi et al.^19,38^, namely that high-temperature curing significantly enhances the early-age strength of concrete but exerts an adverse effect on its later-age strength. Furthermore, the higher the curing temperature, the more significant the loss of later-age strength. The reason is that a higher curing temperature leads to a faster cement hydration rate and a more uneven distribution of hydration products.This results in a higher apparent density of C-S-H gel and less available space for gel packing, which causes coarsening of the internal pore structure of the concrete and consequently a reduction in its later-age strength^28,39^.

### Rate constant and activation energy analysis

**Fig. 2:**
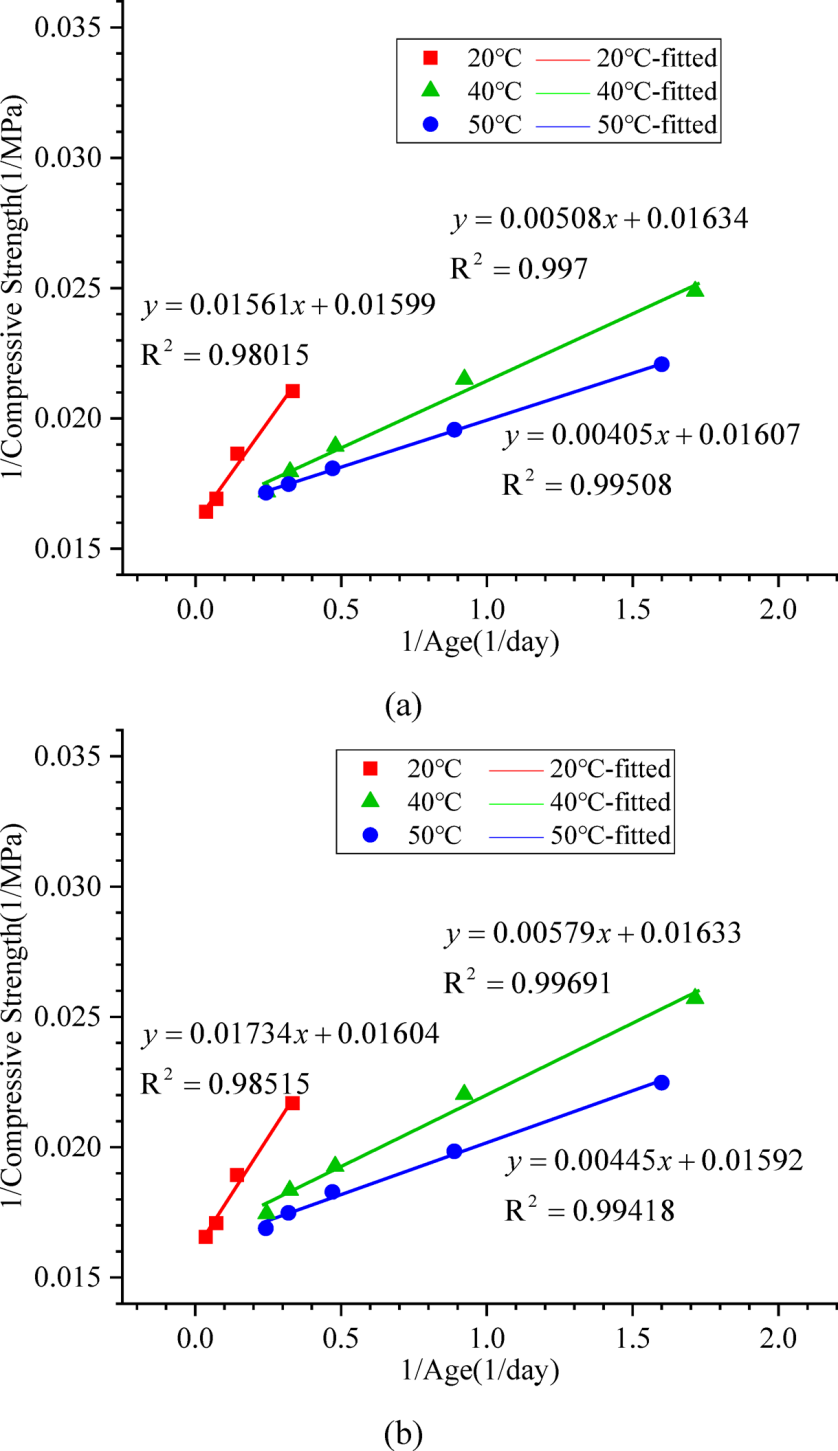
(**a**) Relationship between the reciprocal of strength and the reciprocal of age (manufactured sand concrete). (**b**) Relationship between the reciprocal of strength and the reciprocal of age (natural sand concrete).

Based on the calculation principle for the rate constant (k_T_) of mortar mixtures specified in ASTM C1074^34^, plots of the reciprocal of age against the reciprocal of strength were generated for different curing temperatures, as shown in Fig. 2.

The rate constant k_T_ was calculated using the least squares method, and the results are presented in Table 5.

**Table 5:** Rate constant.

Temp/℃	Natural sand concrete		Manufactured sand concrete	
	k_T_(1/days)	*R* ^2^	k_T_(1/days)	*R* ^2^
20	0.9250	0.98515	1.0244	0.98015
40	2.8204	0.99691	3.2165	0.997
50	3.5775	0.99418	3.9679	0.99508

From Table 5, it can be seen that the rate constants are positively correlated with temperature, which is consistent with previous studies^35,40^.

**Fig. 3:**
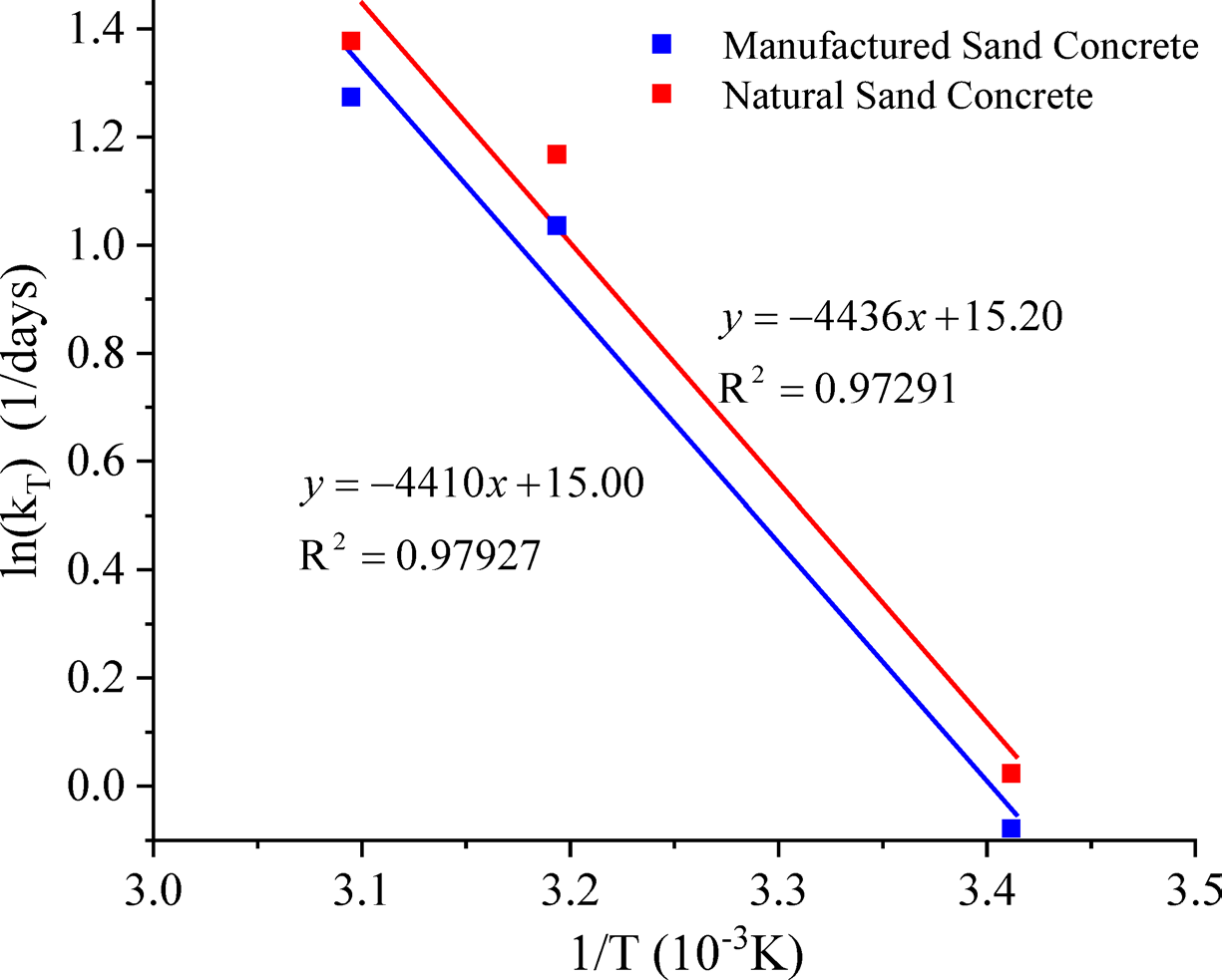
Relationship between ln(k_T_) and 1/T.

Plots of ln(k_T_) against the reciprocal of temperature (1/T, thermodynamic temperature, K) were generated, as shown in Fig. 3.

As can be seen from Fig. 3, the activation energy of manufactured sand concrete is given by E=4436R, and that of natural sand concrete is E=4410R. Using a molar gas constant of *R* = 8.31 J/(mol·K), the activation energies of manufactured sand concrete and natural sand concrete are calculated to be 36,863 J/mol and 36,647 J/mol, respectively. By comparison, the activation energy of C50 manufactured sand concrete is higher than that of C50 natural sand concrete. Therefore, in practical engineering applications, when temperature control measures such as steam curing are adopted for manufactured sand concrete, its relatively high apparent activation energy should be fully taken into account, and the curing regime should be optimized reasonably to ensure the stable development of its compressive strength.

Based on the literature review, Reference^34^ recommends that the activation energy E of ordinary Portland cement concrete ranges from 40,000 to 45,000 J/mol. Poole et al.^41^ used Type I Portland cement (ASTM) with a water-cement ratio of 0.44 and obtained a hydration activation energy of 40,500 J/mol. Barnett et al.^42^ and Turu’allo^43^ investigated the activation energy of concrete with various strength grades (C30, C45, C60 and C75) and measured values ranging from 33,000 to 37,000 J/mol. Overall, the apparent activation energy obtained in this study is generally consistent with the results reported in the existing literature. However, the apparent activation energy E is influenced by numerous factors, including the type of cementitious materials, the degree of hydration, and curing temperature, making it difficult to determine a fixed value for it.

### Relationship between concrete strength and age under standard curing

**Fig. 4:**
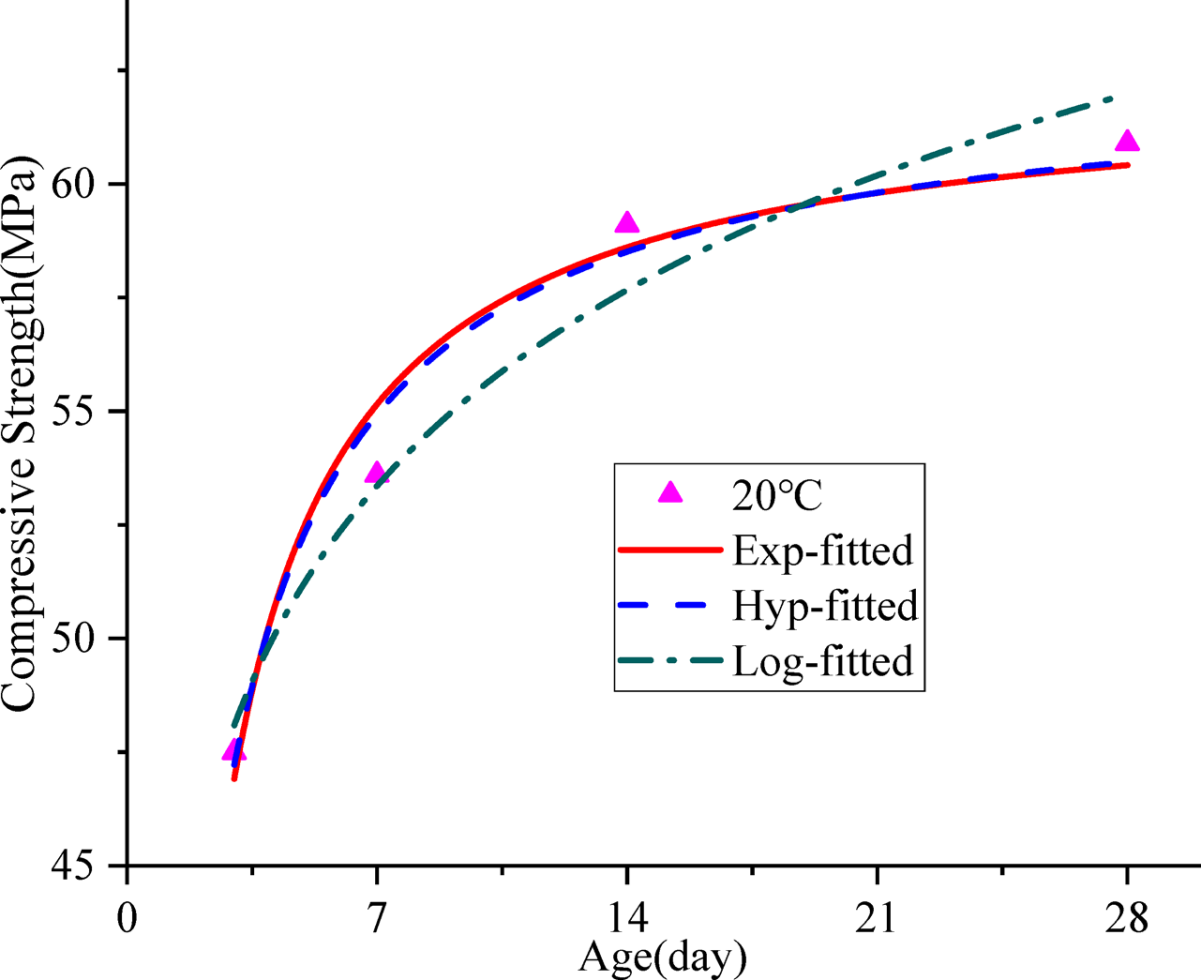
Relationship between concrete strength and age at standard curing.

Based on the compressive strength data of concrete at different ages under standard curing, nonlinear fitting analyses were conducted using Eqs. (5), (6) and (7), as shown in Fig. 4. The relevant fitting parameters are listed in Table 6.

**Table 6:** Regression parameters.

Type	Parameter name	Value	R^2^
$S{\mathrm{=}}{S_u} \cdot \exp {\left[ { - \left( {{\tau \mathord{\left/ {\vphantom {\tau t}} \right. \kern-0pt} t}} \right)} \right]^\alpha }$	S_u_	62.2733	0.97038
	t	0.8943	
	α	0.9500	
$\frac{S}{{{S_u}}}=\frac{{k \cdot \left( {t - {t_0}} \right)}}{{1+k \cdot \left( {t - {t_0}} \right)}}$	S_u_	62.5870	0.97833
	k	1.0256	
	t_0_	0.0041	
$S{\mathrm{=}}a+b \cdot \log {}_{{10}}\left( t \right)$	a	41.2635	0.96696
	b	14.3138	

It can be seen from Table 6 that the exponential, hyperbolic, and logarithmic function models can all describe the relationship between concrete strength and age under standard curing conditions, with the coefficient of determination R^2^ all above 0.96. The hyperbolic function model yields the highest R^2^ value.

### Equivalent age-based strength prediction model for steam-cured manufactured sand concrete

Using a standard curing temperature of 20 °C as the reference temperature, the equivalent ages of the concrete under the three steam curing temperatures were calculated using Eq. (4), with the results presented in Table 7.

**Table 7:** Equivalent age at reference temperature of 20 °C.

40℃		50℃		60℃	
Actual age/h	Equivalent age/h	Actual age/h	Equivalent age/h	Actual age/h	Equivalent age/h
9	13.86	10	18.23	11	23.89
12	19.52	13	25.99	14	34.10
18	31.11	19	41.14	20	54.07
30	53.82	31	71.76	32	94.40
54	99.62	55	133.03	56	175.10
78	145.50	79	194.59	80	256.14
102	191.30	103	255.86	104	336.84

**Fig. 5:**
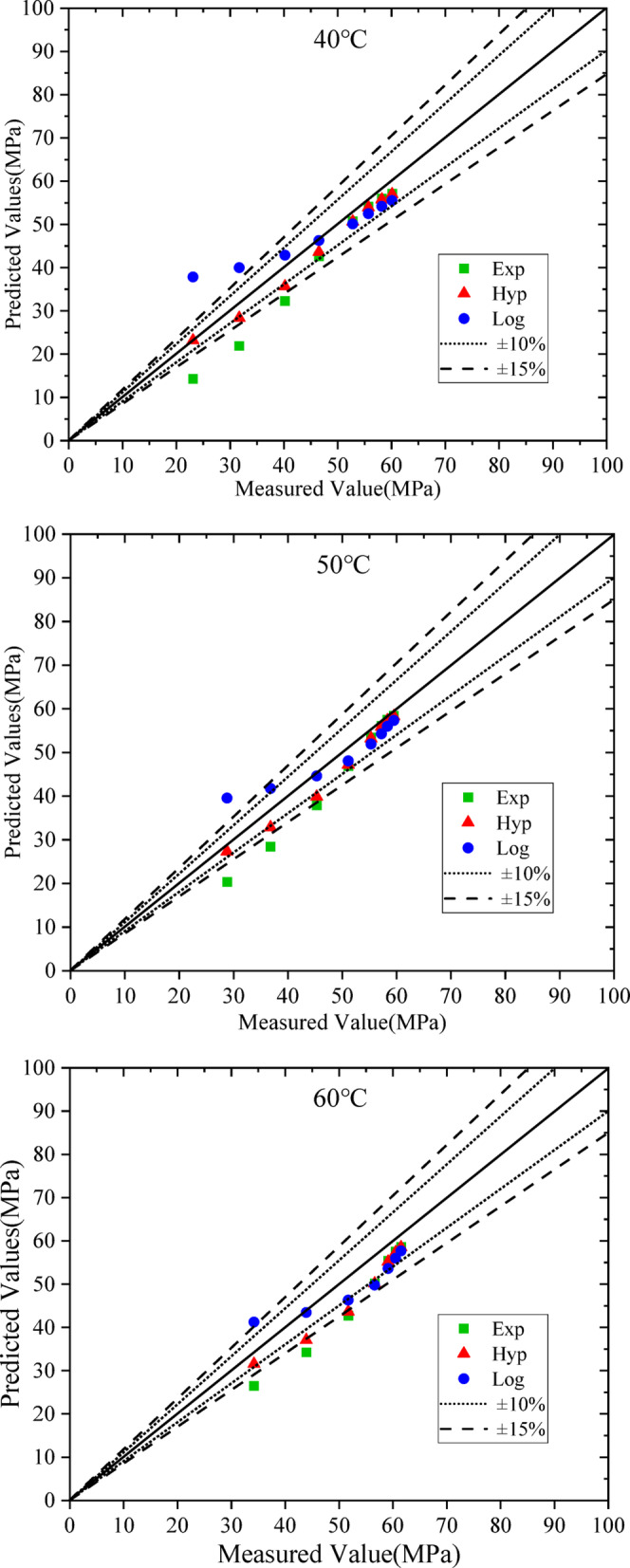
Measured strength versus estimated strength with equivalent age maturity method.

Based on the equivalent age and the regression parameters in Table 6, the compressive strength of concrete at 40 °C, 50 °C and 60 °C were predicted using Eqs. (5), (6) and (7), respectively, and compared with the measured values. The results are shown in Fig. 5; Table 7, where the dotted and dashed lines represent the ± 10% and ± 15% error bands, respectively.

From Fig. 5, for the compressive strength of manufactured sand concrete under steam curing, the hyperbolic and exponential function models underestimate the compressive strength, whereas the logarithmic model overestimates it at early ages and underestimates it at later curing ages. The exponential model yields the largest prediction error, exceeding 15% within the first 14 h for all three steam curing temperatures.

The prediction errors of the hyperbolic model for steam curing at 40 °C and 50 °C are less than 15% over the entire test age, with most predictions within 10%. For steam curing at 60 ℃, the prediction error of the hyperbolic model exceeds 15% between 11 h and 20 h but decreases to less than 10% at later curing ages. Therefore, the hyperbolic model is suitable for predicting the compressive strength of manufactured sand concrete at steam curing temperatures below 50 °C.

At a steam curing temperature of 60 °C, all three models exhibit relatively large prediction errors. This is because an excessively high steam curing temperature accelerates early cement hydration to a great extent, leading to a loose internal microstructure, a deteriorated interfacial transition zone (ITZ), and an increase in microcracks. Consequently, the strength development of the concrete exhibits significant variability, and the applicability of the strength prediction models is significantly reduced under high-temperature curing, which results in larger prediction errors.

## Conclusion

In this paper, C50 manufactured sand concrete was adopted as the research material. The influence of steam curing temperature on its compressive strength was investigated, and the accuracy of the equivalent age‑compressive strength model for predicting the compressive strength of steam-cured manufactured sand concrete was verified. The main conclusions are as follows:

1. Steam curing can greatly shorten the time required for manufactured sand concrete to reach its design strength. However, after a certain curing age, the growth rate of compressive strength slows down significantly, indicating that the effect of steam curing temperature has a certain timeliness. the optimal steam curing durations are 48 h at 40 ℃, 24 h at 50 ℃, and 12 h at 60 ℃.
2. The activation energy E of manufactured sand concrete is 36,863 J/mol, while that of natural sand concrete is 36,647 J/mol; both values fall within the range of apparent activation energy for plain Portland cement concrete without mineral admixtures.
3. The hyperbolic and exponential models underestimate the compressive strength of manufactured sand concrete during the steam curing stage, whereas the logarithmic model overestimates the strength at early curing ages and underestimates it at later curing ages. For manufactured sand concrete at steam curing temperatures below 50 ℃, the prediction errors of the hyperbolic model remain within 15% over the entire test age, demonstrating a higher prediction accuracy than the exponential and logarithmic models.

In future research, a wider range of concrete strength grades will be investigated, and mechanical properties such as the elastic modulus, tensile strength, and flexural strength will be incorporated to further improve the universality and reliability of the research results.

## Data Availability

All data generated or analysed during this study are included in this published article.
